# Supplementary material for: Full 2π tunable phase modulation using avoided crossing of resonances
Source: Nat Commun. 2022 Apr 19;13:2103. doi: 10.1038/s41467-022-29721-7 (PMC9018797; doi:10.1038/s41467-022-29721-7)
Supplement: Supplementary file 1 — Supplementary Information [file 41467_2022_29721_MOESM1_ESM.pdf]

Supplementary Information for

Full 2π Tunable Phase Modulation Using Avoided Crossing of Resonances

Ju Young Kim[1]†, Juho Park[1]†, Gregory R. Holdman[2], Jacob T. Heiden[1], Shinho Kim[1], Victor W. Brar[2]*, and Min Seok Jang[1]*

[1]School of Electrical Engineering, Korea Advanced Institute of Science and Technology, Daejeon 34141, Republic of Korea

[2]Department of Physics, University of Wisconsin-Madison, Madison, Wisconsin 53706, United States

Correspondence to:
* vbrar@wisc.edu
* jang.minseok@kaist.ac.kr

†These authors contributed equally to this work.

This PDF file includes:

Supplementary Text
Supplementary Figs. 1 to 5
Supplementary Table 1
References

Supplementary Note 1. Radiative and dissipative loss rates of the metasurface

Following the formalism of the TCMT (temporal coupled mode theory) section below, we can extract the values of the qBIC (quasi-bound states in the continuum) radiative loss rate and its dissipative loss rate respectively, from full wave electromagnetic simulation results of a setup with a graphene sheet deposited through the entire unit cell underneath the Si pillars. In this setup graphene plasmons will be off-resonance and too weak to play a role, therefore satisfying the condition of a singular resonance (the qBIC) system and simplifying the equations down to Supplementary Eq. 1, discussed in Supplementary Note 3. The extracted radiative loss rates and the dissipative loss rates of the qBIC depending on the metasurface structural changes are shown in Supplementary Fig. 1. We can note that the structural changes do not largely affect dissipative loss rates, whereas they heavily influence radiative loss rates.

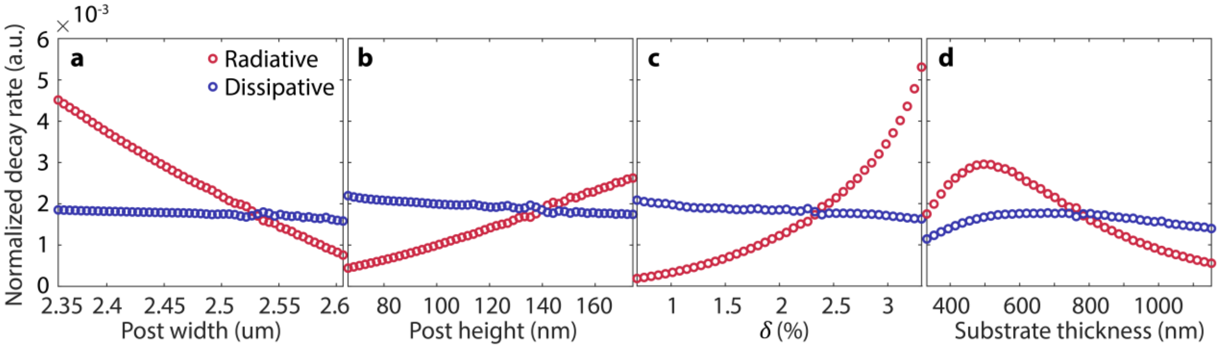

Supplementary Figure 1. The dependence of the qBIC's radiative and dissipative loss rates with respect to structural parameters. The dependence with respect to **a,** the Si pillar post width **b,** the post height **c,** the δ and **d,** the substrate thickness. The dissipative loss rates are not largely affected by the changes in the structural geometry, whereas the radiative coupling is heavily influenced by the structural changes due to the corresponding changes in the strengths of symmetry-breaking-induced dipole moments.

1.1 Radiative loss rates

The radiative loss is largely affected because it is dependent on the strength of the dipole moment (allowing coupling to free-space radiation) that is formed due to the dimerization (symmetry-breaking) of the metasurface, and this is greatly influenced by the structural changes. For example, raising the height of the Si pillars would increase the amount of charge accumulation that leads to a dipole formation, resulting in a larger radiative coupling. Increasing post width will decrease the central gap, leading to a shorter dipole length/smaller dipole moment and therefore a lower radiative loss rate. The dimerizing perturbation δ is defined to express the percentage of the shift

of the center of the Si pillars in the direction of dimerization, and the magnitude of the shift is defined by $\delta\Lambda/2$. The dimerizing perturbation δ affects the degree of the perturbation (symmetry breaking/dimerization) and a larger delta would correspond to a larger dipole moment, resulting in a larger radiative coupling. This relation is shown to be linear in the limit of smaller perturbation and non-linear as the perturbation gets larger (*33*). The substrate thickness changes the degree of the Fabry-Perot effect between the back-reflector and the Si pillars, and the radiative coupling draws a rough sinusoidal shape as the dipole moment is affected by the constructive/destructive interference with the light reflected from the back reflector.

1.2 Dissipative loss rates

The dissipative loss rate, on the other hand, is not majorly affected by structural changes but instead greatly on the changes in the graphene Fermi level. This is shown in Supplementary Fig. 2a, where the initial hump of the dissipative loss rate near 0 eV is due to the interband transition losses and the steady increase from 0.2 eV is due to the changes in the intraband transition losses. The resonance frequency of the qBIC is roughly 41 THz, which would mean that at this frequency interband transition losses would be cut off from around roughly 0.08-0.09 eV due to Pauli blocking and the corresponding change in the graphene's optical conductivity (*27*). This explains the drop in the dissipative loss rate from 0 eV to 0.2 eV. Then the intraband scattering in graphene slowly increases as the Fermi level increases due to the rise in the carrier density and an increase in the optical conductivity (*27*), which explains the steady increase in the dissipative loss rate.

We can employ graphene plasmons as well as the qBIC by depositing graphene ribbons between the Si pillars, as opposed to a whole graphene sheet underneath the Si pillars throughout the unit cell. In this case, the coupling between the qBIC and the graphene plasmon affects the radiative and the dissipative loss rates and therefore the extracted loss rate values from using a single resonance TCMT model becomes unreliable. Using the two-resonance TCMT model explained below would entail too many parameters at once and the extraction of the loss rate values would be unviable as well. This is shown in Supplementary Fig. 2b, where the high coupling zone region from 0.5-0.8 eV is denoted by a grey box. The loss rate values for the qBIC shows a similar trend to that of Supplementary Fig. 2a, with slight differences in the high Fermi level range due to the presence of graphene plasmons occurring in the side graphene ribbons. This can be seen in Fig. 3e of the main paper, where GP_2 (the secondary graphene plasmon) approaches the qBIC line and

further broadens the linewidth. This would technically introduce errors to our loss rate extraction method based on the single resonance TCMT model explained below. The loss rates for the graphene plasmon are shown as well, with the dissipative loss rate nicely following the DC scattering rate $1/\tau = ev_F^2/E_F\mu$ where e is the electron charge, μ is the mobility currently set at 1000 $cm^2/(V\cdot s)$, and $v_F \approx c/300$ is the Fermi velocity (*42*). The graphene plasmon is a dipole radiator as can be seen Fig. 4c of the main paper, and radiation from a dipole radiator increases as the frequency of the radiation increases (*43*). Because the resonance frequency of the graphene plasmon drastically increases as the Fermi level is increased (Fig. 3e of the main paper), its radiative loss rate also increases until the high coupling zone.

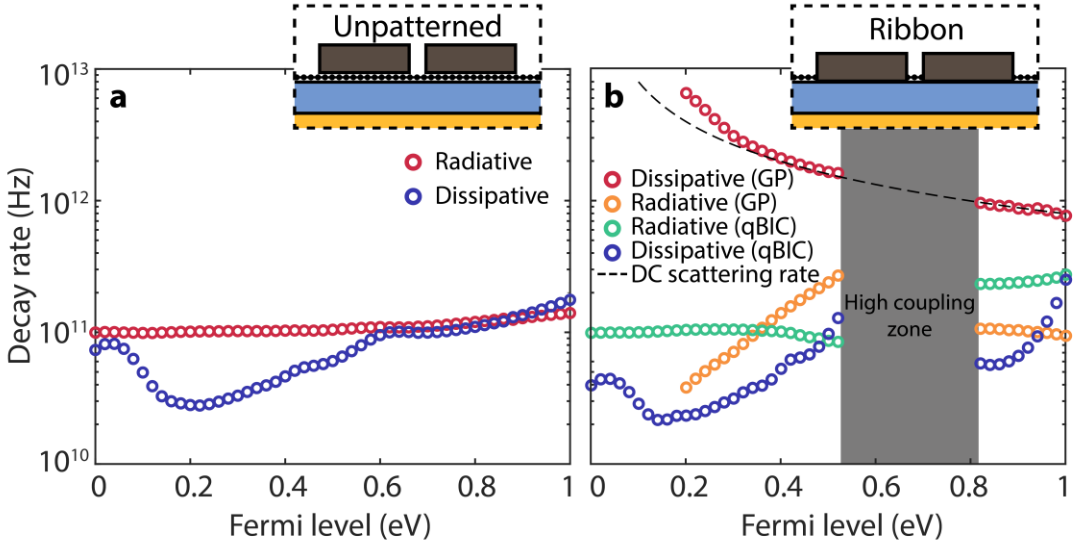

Supplementary Figure 2. The dependence of the radiative and dissipative loss rates with respect to the graphene Fermi level. a, The qBIC's radiative loss rate is not largely affected by the changes in the graphene fermi level, whereas it heavily influences the dissipative loss rate due to the interband/intraband scattering in graphene. **b,** Dissipative loss rates for both the qBIC and graphene plasmons (employed through graphene ribbons as opposed to a graphene sheet in **a**). The qBIC's radiative and dissipative loss rates follow a similar trend to that in **a** with the coupling between the qBIC and the graphene plasmon, as well as the effect of a secondary graphene plasmon from the side graphene ribbons, playing a significant role in the higher fermi levels close to 1 eV.

Supplementary Note 2. Structure parameter optimization

Since the optical response of our graphene - Si structure involves complicated dynamics of both qBIC and graphene plasmon resonances, the operating wavelength and the corresponding geometric parameters have to be numerically optimized and fine-tuned. For this purpose, we employed Reticolo RCWA (rigorous coupled-wave analysis) software (*44,45*) and the gradient-free algorithm BOBYQA (bound optimization by quadratic approximation) (*46,47*) to obtain the exact geometric parameters for maximal phase modulation.

First, we defined a figure of merit (FoM) in order to quantify the phase modulation performance. The FoM was defined as the area enclosed by the complex reflectivity curve drawn on the complex plane as the graphene Fermi level being tuned at a single target frequency. This definition facilitated with the simultaneous optimization of both the reflection amplitude and the phase coverage.

Second, we utilized the gradient-free algorithm BOBYQA for the structural parameter optimization. Gradient-free algorithms are suitable for problems in which calculating the objective function gradient over the design variable space is infeasible or impossible. Our structure exhibiting complicated dynamics with respect to changes in geometry that is analytically intractable, making gradient-free algorithm a preferred optimization algorithm over gradient-based algorithms.

To complete the optimization program we had to find a suitable simulation tool for our structure. We found that RCWA method was proper for this task, since its simulation times are much faster than that of FDTD (finite-difference time-domain) or FEM (finite element method) methods, while providing satisfactory accuracy. For the graphene sheet case, the Fourier order needed to reproduce similar reflectance spectra to FEM results was only 100, for which evaluating 51 Fermi energy points took less than 5 seconds on our server computer (Intel E5-2680v4). However, for the graphene ribbon case, the required Fourier order was about 600, which took roughly 200 times longer than that of the case with 100 Fourier orders. The reason for such a long computation time stems from the extremely narrow width of the graphene nanoribbons compared to the structure period. In order to bypass this problem, we first optimize geometric structural parameters for a maximum FOM at low Fourier orders, and then set the obtained geometric parameters as an initial

point for the next optimization procedure with higher Fourier orders. This gradual increase of the Fourier orders rendered the simulation more accurate.

Finally, the simulation results generated by the RCWA was compared to that of the commercial FEM tool (COMSOL Multiphysics), which demonstrated excellent agreement.

Supplementary Note 3. Temporal coupled mode theory (TCMT)

From (*48*), it is shown that the governing equation for a system with two resonances is

$$\frac{\mathrm{d}\mathbf{a}}{\mathrm{d}t} = (-i\mathbf{\Omega} - \mathbf{\Gamma})\mathbf{a} + \mathbf{K}s_+$$

$$s_- = Cs_+ + \mathbf{D}\mathbf{a}$$

Since our system has only one radiative channel, s_+ (input), s_- (output), and C are scalar. Eigenmode amplitudes (quasi-BIC and graphene plasmon resonance) are expressed as $\mathbf{a} = \begin{pmatrix} a_1 \\ a_2 \end{pmatrix}$, and the matrix terms are given as follows:

$$\mathbf{\Omega} = \begin{pmatrix} \omega_1 & u \\ u & \omega_2 \end{pmatrix}, \qquad \mathbf{\Gamma} = \begin{pmatrix} \gamma_{1r} & \gamma_{12} \\ \gamma_{21} & \gamma_{2r} \end{pmatrix}$$

Here, ω_1 and ω_2 are the resonance frequencies and γ_{1r} and γ_{2r} are the radiative decay rates of the modes. The off-diagonal terms describe the coupling between the modes. For now, we assume that there is no dissipative loss.

$$\mathbf{K} = \begin{pmatrix} k_1 \\ k_2 \end{pmatrix}, \qquad \mathbf{D} = (d_1 \quad d_2)$$

K describes the coupling from the input channel (s_+) to the resonant modes, while **D** describes the coupling from the resonant modes to the output channel (s_-).

From the following conditions, the coefficients can be determined as follows:

(i) By energy conservation, $\mathbf{D}^\dagger\mathbf{D} = \mathbf{2\Gamma}$:

$$\begin{pmatrix} d_1^* d_1 & d_1^* d_2 \\ d_2^* d_1 & d_2^* d_2 \end{pmatrix} = \begin{pmatrix} 2\gamma_{1r} & 2\gamma_{12} \\ 2\gamma_{21} & 2\gamma_{2r} \end{pmatrix} \rightarrow \begin{cases} |d_{1,2}| = \sqrt{2\gamma_{1,2r}} \\ \gamma_{12} = \gamma_{21}^* = \sqrt{\gamma_{1r}\gamma_{2r}} e^{i(\theta_2 - \theta_1)} \end{cases}$$

where θ_1 and θ_2 are the arguments of d_1 and d_2, respectively.

(ii) By reciprocity, $\mathbf{K}^\mathrm{T} = \mathbf{D}$ and $C\mathbf{D}^* = -\mathbf{D}$:

$$k_{1,2} = d_{1,2} = \sqrt{2\gamma_{1,2r}} e^{i\theta_{1,2}}$$

$$C \begin{pmatrix} \sqrt{2\gamma_{1r}} e^{-i\theta_1} \\ \sqrt{2\gamma_{2r}} e^{-i\theta_2} \end{pmatrix}^\mathrm{T} = -\begin{pmatrix} \sqrt{2\gamma_{1r}} e^{i\theta_1} \\ \sqrt{2\gamma_{2r}} e^{i\theta_2} \end{pmatrix}^\mathrm{T} \rightarrow C = -e^{2i\theta_1} = -e^{2i\theta_2} \rightarrow e^{i(\theta_2 - \theta_1)} = \pm 1$$

Therefore, $\gamma_{12} = \gamma_{21} = \pm\sqrt{\gamma_{1r}\gamma_{2r}}$.

Small material losses (γ_{1d} and γ_{2d}) can be included perturbatively as

$$\mathbf{\Gamma} = \begin{pmatrix} \gamma_{1r} + \gamma_{1d} & \pm\sqrt{\gamma_{1r}\gamma_{2r}} \\ \pm\sqrt{\gamma_{1r}\gamma_{2r}} & \gamma_{2r} + \gamma_{2d} \end{pmatrix}$$

At a steady state under illumination of light at a fixed frequency ω,

$$-i\omega\mathbf{I}\mathbf{a} = (-i\mathbf{\Omega} - \mathbf{\Gamma})\mathbf{a} + \mathbf{K}s_+ \rightarrow \mathbf{a} = i(\omega\mathbf{I} - \mathbf{\Omega} + i\mathbf{\Gamma})^{-1}\mathbf{K}s_+$$

$$s_- = Cs_+ + \mathbf{D}\mathbf{a} = Cs_+ + i\mathbf{D}(\omega\mathbf{I} - \mathbf{\Omega} + i\mathbf{\Gamma})^{-1}\mathbf{K}s_+ \rightarrow r = \frac{s_-}{s_+} = C + i\mathbf{D}(\omega\mathbf{I} - \mathbf{\Omega} + i\mathbf{\Gamma})^{-1}\mathbf{D}^{\mathrm{T}}$$

$$r = e^{i2\theta_1}\left[-1 + 2i\frac{\gamma_{1r}(\omega - \omega_2 + i\gamma_{2d}) + \gamma_{2r}(\omega - \omega_1 + i\gamma_{1d}) \pm 2u\sqrt{\gamma_{1r}\gamma_{2r}}}{\big(\omega - \omega_1 + i(\gamma_{1r} + \gamma_{1d})\big)\big(\omega - \omega_2 + i(\gamma_{2r} + \gamma_{2d})\big) - (u \mp i\sqrt{\gamma_{1r}\gamma_{2r}})^2}\right]$$

Note that, $\phi_0 = \pi - 2\theta_1$ is the phase shift upon reflection in the absence of resonances.

Similarly, for a single resonance with a single radiation channel, the complex reflection amplitude is given by (ignoring the overall phase shift):

$$r = -1 + 2i\frac{\gamma_{1r}}{\big(\omega - \omega_1 + i(\gamma_{1r} + \gamma_{1d})\big)}$$

$$= \frac{(\gamma_{1r} - \gamma_{1d}) - i(\omega - \omega_1)}{(\gamma_{1r} + \gamma_{1d}) + i(\omega - \omega_1)} \quad \text{(Supplementary Eq. 1)}$$

This leads to the reflectance and the FWHM:

$$R = |r|^2 = 1 - 4\frac{\gamma_r\gamma_d}{(\gamma_r + \gamma_d)^2} = 1 - \frac{4p}{(p+1)^2} \quad p = \frac{\gamma_r}{\gamma_d}$$

$$\mathrm{FWHM} = 2(\gamma_r + \gamma_d)$$

Supplementary Note 4. Comparison of the theoretical TCMT model and simulation results

With the analytical two-resonance model for the complex reflection amplitude r, we can compare the theoretical reflectance spectra for an avoided crossing with two resonances and that of the simulation obtained from full wave simulations (Fig. 3 of the main paper). To model the resonance frequency of the qBIC as a function of fermi level, we assumed that the qBIC resonance follows a linear trend due to the perturbative change of the graphene permittivity with respect to a much larger qBIC mode volume spanned around the Si pillars. The fitting parameters were chosen as $\omega_{qBIC} = c_1 E_F + c_2$ with $c_1 = 0.4188\ \mathrm{THz/eV}$ and $c_2 = 41.1351\ \mathrm{THz}$ to fit the full wave simulation results. For the graphene plasmon it is known that $\omega_{GP} \propto \sqrt{E_F}$ (*27*), and to fit it to the simulation results we set $\omega_{GP} = c_3\sqrt{E_F}$ with $c_3 = 52.65\ \mathrm{THz/eV^{1/2}}$.The coupling constant u in the TCMT section above was taken to be $u = -1.03\ \mathrm{THz}$ to fit the simulation results. For the qBIC's radiative loss rate we took the constant value of 10^{11} Hz from Supplementary Fig. 2a, and for the dissipative loss rate we took the data from Supplementary Fig. 2a and multiplied a constant of 0.539 to equalize the value to that of Supplementary Fig. 2b at the fermi level 0 eV (the furthest away from the high coupling zone and the secondary graphene plasmon effects). For the graphene plasmon's dissipative loss rate we used the DC scattering rate $1/\tau = ev_F^2/E_F\mu$ with the corresponding parameters. For its radiative loss rate we took the average of 3×10^{11} Hz at the beginning of the high coupling zone and 1×10^{11} Hz at the end of the high coupling zone and used the value of 2×10^{11} Hz, because it's within the high coupling zone that the effects of avoided crossing would dominantly occur.

Supplementary Note 5. Device performance and the spectral line-cuts

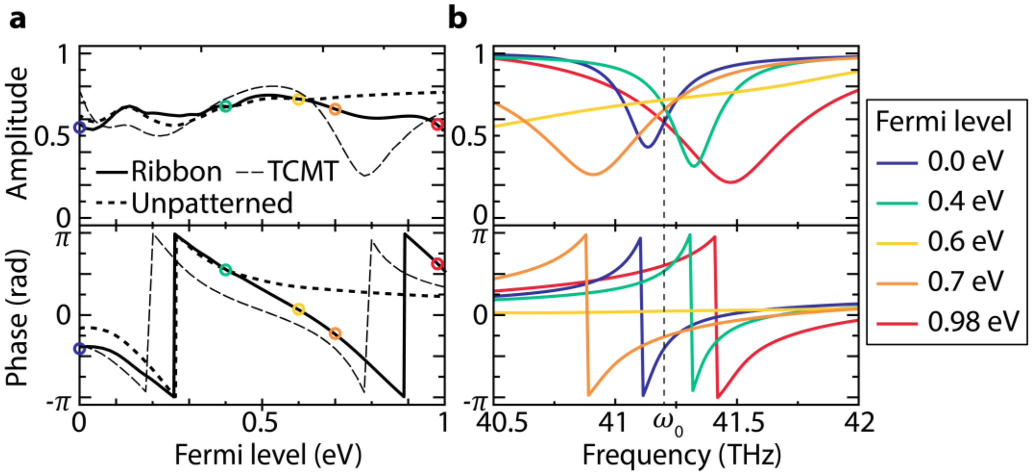

Supplementary Figure 3. Device performance and the spectral line-cuts. a, The amplitude and phase of the graphene ribbon structure with the avoided crossing, the theoretical TCMT case with the avoided crossing, and the unpatterned graphene case without the avoided crossing. **b,** The spectral line-cuts of the 5 notable Fermi level values for the graphene ribbon structure case that illustrate how the two resonances sweep across the operating frequency.

Supplementary Note 6. Phase modulation performance dependence on the TCMT parameters

We investigated whether it was better performance-wise for the spectrally narrow resonance to have the smallest FWHM as possible and for the second resonance to have the greatest $\Delta\omega$ as possible. In general, the decreasing FWHM of the spectrally narrow resonance (denoted as Resonance 1 hereafter) with respect to $\Delta\omega$ of the second resonance (denoted as Resonance 2 hereafter) leads to a better device performance (See Fig. 1b main text). However, there exist a few additional requirements that must be satisfied simultaneously:

- **Resonance 1 should be highly over-coupled ($\gamma_{1r} \gg \gamma_{1d}$)**: The FWHM of Resonance 1 can be decomposed to $FWHM_1 = 2(\gamma_{1r} + \gamma_{1d})$, where γ_{1r} and γ_{1d} are the radiative and dissipative coupling rate of Resonance 1. Even if the $FWHM_1$ is very small, if Resonance 1 is under-coupled ($\gamma_{1r} < \gamma_{1d}$) the device will fail to cover the range 0-2π in phase modulation as shown in Fig.1a and Supplementary Fig. 4b. A decrease in γ_{1d} while γ_{1r} is kept constant, on the other hand, is beneficial not only because it decreases the FWHM_1 but also increases the ratio $p = \gamma_r/\gamma_d$, thereby increasing the reflection amplitude through $R = |r|^2 = 1 - 4p/(p+1)^2$ and making it more uniform as shown in Supplementary Fig. 4c. It is evident that the complex reflection amplitude draws a larger phase modulation circle while having a more uniform radius in Supplementary Fig. 4c than the reference case in Supplementary Fig. 4a.
- **γ_{1r} should not be too small compared to γ_{2d}**: Even if it's ensured that Resonance 1 stays highly over-coupled ($\gamma_{1r} \gg \gamma_{1d}$), decreasing γ_{1r} does not always bring performance enhancement. If γ_{1r} is much smaller than γ_{2d} of Resonance 2 in terms of their order of magnitudes ($\gamma_{1r} \ll \gamma_{2d}$), the phase modulation will fail to cover the range 0-2π as shown in Supplementary Fig. 4d, a case where both γ_{1r} and γ_{1d} are decreased while keeping γ_{2d} the same as the reference. This is because the hybridized mode (with the characteristics of having both γ_{1r} and γ_{2d}) will be too under-coupled near the avoided crossing region and the phase modulation will fail to circle around the origin.
- **Resonance 2 should be highly tunable ($\Delta\omega_2 >$ FWHM$_1$)**: Having a greater $\Delta\omega_2$ will lead to a better phase modulation performance. This is because the hybridized modes will cross the operating frequency in greater speed as the control parameter (in our case the Fermi level) is varied. This is illustrated in Supplementary Fig. 4e.

From the discussions above, we can combine the different cases and plot the ideal case of having large $\Delta\omega_2$ and small $\gamma_{1r}, \gamma_{1d}, \gamma_{2d}$ with $\gamma_{1r} \gg \gamma_{1d}$ & $\gamma_{1r} > \gamma_{2d}$, as shown in Supplementary Fig. 4f.

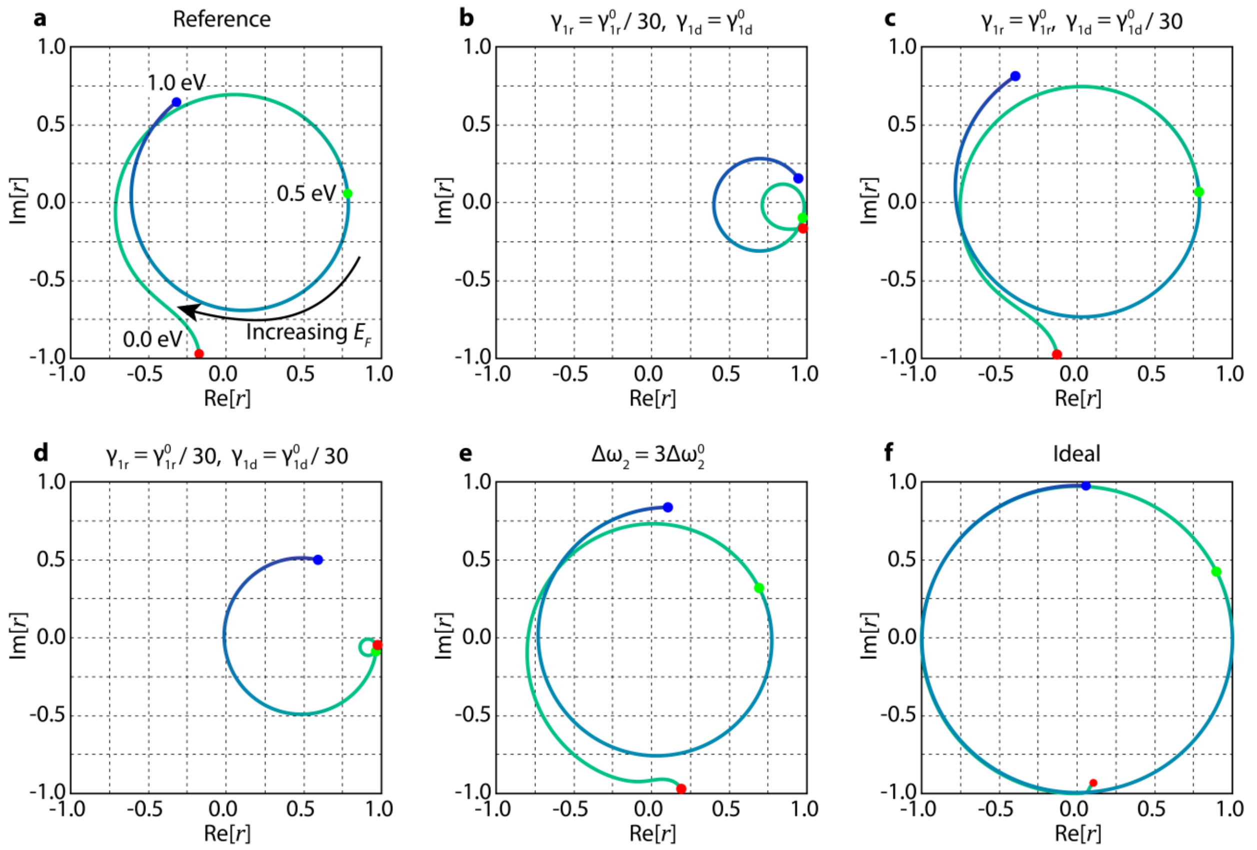

Supplementary Figure 4. Dependence of the phase modulation performance with respect to different TCMT parameters shown in Supplementary Table 1. a, The complex reflection amplitude for the reference TCMT parameter case. **b,** The under-coupled case where $\gamma_{1r} = \gamma_{1r}^0/30$ and $\gamma_{1d} = \gamma_{1d}^0$, with other parameters kept the same. The details are shown in Table S1 below. **c,** The case where $\gamma_{1r} = \gamma_{1r}^0$ and $\gamma_{1d} = \gamma_{1d}^0/30$. **d,** The case where $\gamma_{1r} = \gamma_{1r}^0/30$ and $\gamma_{1d} = \gamma_{1d}^0/30$ with $\gamma_{2d} = \gamma_{2d}^0$. **e,** The case where $\Delta\omega_2 = 3\Delta\omega_2^0$. **f,** The ideal case where $\gamma_{1r} = \gamma_{1r}^0$, $\gamma_{1d} = \gamma_{1d}^0/30$, $\gamma_{2d} = \gamma_{2d}^0/30$ and $\Delta\omega_2 = 3\Delta\omega_2^0$.

Case	$\Delta\omega_2$	γ_{1r}	γ_{1d}	γ_{2d}
Reference (Supplementary Fig. 4A)	$\Delta\omega_2^0$	γ_{1r}^0	γ_{1d}^0	γ_{2d}^0
Undercoupled qBIC (Supplementary Fig. 4B)	$\Delta\omega_2^0$	$\gamma_{1r}^0/30$	γ_{1d}^0	γ_{2d}^0
Small γ_{1d} (Supplementary Fig. 4C)	$\Delta\omega_2^0$	γ_{1r}^0	$\gamma_{1d}^0/30$	γ_{2d}^0
Small γ_{1r}, Small γ_{1d} (Supplementary Fig. 4D)	$\Delta\omega_2^0$	$\gamma_{1r}^0/30$	$\gamma_{1d}^0/30$	γ_{2d}^0
Large $\Delta\omega_2$ (Supplementary Fig. 4E)	$3\Delta\omega_2^0$	γ_{1r}^0	γ_{1d}^0	γ_{2d}^0
Ideal (Supplementary Fig. 4F)	$3\Delta\omega_2^0$	γ_{1r}^0	$\gamma_{1d}^0/30$	$\gamma_{2d}^0/30$

Supplementary Table 1. The numerical values/functions of the TCMT parameters used in Supplementary Fig. 4.

Supplementary Note 7. Relocation of the avoided crossing for limited Fermi level tuning range

The device presented in the main paper can only cover 282° for E_F = 0-0.6eV as shown in Fig. 3d. The reason for this sub-2π phase coverage is that the device utilizes only one resonance in this E_F range since the avoided crossing between the graphene plasmon resonance and qBIC mode occurs at the upper bound of the E_F range (E_F = 0.6 eV). As mentioned before, achieving full 2π coverage requires having a double crossover between the operating frequency line and the two hybrid modes. Therefore, to have a better phase coverage with a limited E_F tuning range, the device parameters should be altered to have the avoided crossing at a lower Fermi level. This can be done by either narrowing the center graphene width to obtain the GP resonance at a lower Fermi energy or by increasing the meta-atom period to lower the frequency of the qBIC resonance. The other design parameters, which determines the radiative and dissipative decay rates of the resonances, also need to be fine-tuned to achieve a nearly uniform reflection amplitude.

To test this possibility, we conducted an optimization for 0-0.6eV Fermi level range as shown in Supplementary Fig. 5. Compared to the structure presented in the main paper (blue line in Supplementary Fig. 5a), the low-E_F-optimized device (red line in Supplementary Fig. 5a) has a 15.6% longer period Λ but the gap between the Si pillars is nearly the same for the two structures. As shown in Supplementary Fig. 5a, the reflection curve of the low-E_F-optimized device can cover full 2π phase change while its magnitude is slightly lower than the curve in Fig. 3d. Note that, as shown in Supplementary Fig. 5b, c, the avoided crossing occurs at around E_F = 0.5 eV, which is included in the 0-0.6 eV tuning range.

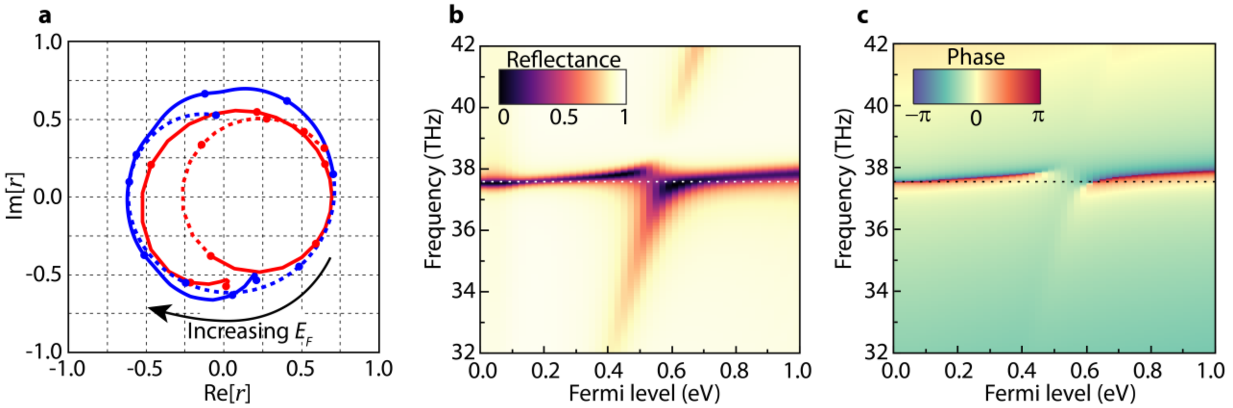

Supplementary Figure 5. **Full wave calculations of the reflected light for a dimerized Si grating with a relocated avoided crossing, optimized for a limited Fermi level tuning range. a,** The complex reflection amplitude plots for the structure presented in the main paper and a structure optimized for a limited E_F range of E_F = 0-0.6eV. The parameters are: period Λ = 6155 nm, h = 61 nm, d = 896 nm, w = 888 nm, δ = 34.9 %, center gap = 38.7 nm, side gap = 4340 nm, and operating frequency = 37.64 THz. **b, c,** The reflectance and the phase color plot, respectively, for the new structure. The operating frequency is shown as the dotted line.

References

42. Stauber, T., Peres, N. M. R., Guinea, F. Electronic transport in graphene: A semiclassical approach including midgap states. *Phys. Rev. B* **76**, 205423 (2007).

43. Jackson, J. D. *Classical electrodynamics* (Wiley, 1998).

44. Moharam, M. G., Grann, E. B., Pommet, D. A., Gaylord, T. K. Formulation for Stable and Efficient Implementation of the Rigorous Coupled-Wave Analysis of Binary Gratings. *J. Opt. Soc. Am. A* **12**, 1068-1076 (1995).

45. Hugonin, J. P., Lalanne, P. RETICOLO software for grating analysis, arXiv:2101.00901 https://arxiv.org/abs/2101.00901 (2021).

46. Powell, M. J. D. The BOBYQA algorithm for bound constrained optimisation without derivatives, Report No. DAMTP 2009/NA06, University of Cambridge, UK, (2009).

47. Cartis, C., Fiala, J., Marteau, B., Roberts, L. Improving the Flexibility and Robustness of Model-based Derivative-free Optimization Solvers. *ACM T. Math. Software* **45**, 32 (2019).

48. Suh, W. W, Wang, Z., Fan, S. Temporal coupled-mode theory and the presence of non-orthogonal modes in lossless multimode cavities. *IEEE J. Quantum Electron* **40**, 1511-1518 (2004).
